# Association of Adiponectin SNP+45 and SNP+276 with Type 2 Diabetes in Han Chinese Populations: A Meta-Analysis of 26 Case-Control Studies

**DOI:** 10.1371/journal.pone.0019686

**Published:** 2011-05-11

**Authors:** Yiping Li, Xianli Li, Li Shi, Man Yang, Ying Yang, Wenyu Tao, Lei Shi, Yuxin Xiong, Ying Zhang, Yufeng Yao

**Affiliations:** 1 Department of Endocrinology and Metabolism, The Second People's Hospital of Yunnan Province, Kunming, Yunnan, China; 2 Institute of Medical Biology, Chinese Academy of Medical Sciences & Peking Union Medical College, Kunming, Yunnan, China; University of Las Palmas de Gran Canaria, Spain

## Abstract

Recently, many studies have reported that the SNP+45(T>G) and SNP+276(G>T) polymorphisms in the adiponectin gene are associated with type 2 diabetes (T2DM) in the Chinese Han population. However, the previous studies yielded many conflicting results. Thus, a meta-analysis of the association of the adiponectin gene with T2DM in the Chinese Han population is required. In the current study, we first determined the distribution of the adiponectin SNP+276 polymorphism in T2DM and nondiabetes (NDM) control groups. Our results suggested that the genotype and allele frequencies for SNP+276 did not differ significantly between the T2DM and NDM groups. Then, a meta-analysis of 23 case-control studies of SNP+45, with a total of 4161 T2DM patients and 3709 controls, and 11 case-control studies of SNP+276, with 2533 T2DM patients and 2212 controls, was performed. All subjects were Han Chinese. The fixed-effects model and random-effects model were applied for dichotomous outcomes to combine the results of the included studies. The results revealed a trend towards an increased risk of T2DM for the SNP+45G allele as compared with the SNP+45T allele (OR = 1.34; 95% CI, 1.11–1.62; P<0.01) in the Chinese Han population. However, there was no association between SNP+276 and T2DM (OR = 0.90; 95% CI, 0.73–1.10; P = 0.31). The results of our association study showed there was no association between the adiponectin SNP+276 polymorphism and T2DM in the Yunnan Han population. The meta-analysis results suggested that the SNP+45G allele might be a susceptibility allele for T2DM in the Chinese Han population. However, we did not observe an association between SNP+276 and T2DM.

## Introduction

Diabetes mellitus comprises a group of metabolic disorders that are characterized by chronic hyperglycemia. Type 2 diabetes (T2DM) and its complications impose a tremendous burden both on individuals with diabetes and on the health care system.

Adiponectin is an important adipocytokine that is secreted by adipocytes and plays a key role in the regulation of insulin sensitivity and glucose homeostasis[1], [2], [3], [4]. Serum adiponectin concentrations are decreased in patients with T2DM, obesity, and metabolic syndrome[4], [5], [6], [7]. Many studies have reported that single nucleotide polymorphisms (SNPs) in the adiponectin gene are associated with T2DM in different populations[8], [9], [10], [11], [12], [13], [14]. However, the results of these studies are confusing rather than conclusive, and show strong racial and regional variations [11], [13], [15], [16], [17], [18], [19], [20].

The Chinese population comprises 56 ethnic groups, of which Han is the largest (approximately 93% of the total population). The Han are descended from the Huaxia tribe who lived in the area of the Yellow River, and then migrated to the area of the Yangtze River. The Han have now spread throughout the country and live in different regions of China. Many studies that investigated the association in the Chinese Han population between T2DM and SNPs in the adiponectin gene have been reported recently [16], [19], [20], [21], [22], [23], [24], [25], [26], [27], [28], [29], [30], [31], [32], [33], [34], [35], [36], [37], [38], [39], [40], [41], [42], [43]. Two SNPs in particular have been analyzed: rs2241766 (+45 T>G in exon 2) and rs1501299 (+276 G>T in intron 2). A series of association studies was carried out to investigate the relationship between these two SNPs and T2DM in the Han population in different regions of China. However, the results were still fairly contradictory, and showed regional variation. Therefore, a meta-analysis of the association of the adiponectin gene polymorphisms SNP+45 and SNP+276 with T2DM in the Chinese Han population is necessary in order to clarify the role of these SNPs in T2DM.

The aim of the current study was first to evaluate the association of SNP+276 with T2DM in the Han population in Yunnan Province, in the southwest of China. Then, we combined our data with those of 25 previous studies for a carefully designed meta-analysis to investigate the association of the SNP+45 and SNP+276 polymorphisms in the adiponectin gene with T2DM in the Chinese Han population. Our study will provide a more thorough evaluation of the significance of the association of the SNP+45 and SNP+276 polymorphisms with T2DM in the Chinese Han population.

## Results

### 1. Association study results

#### 1.1 Clinical characteristics of the enrolled subjects

The clinical characteristics of the enrolled subjects are presented in Table S1. Anthropometric parameters (BMI, WC, and HC) were significantly higher in patients with T2DM than in NDM subjects. Although fasting insulin did not differ between the T2DM and NDM groups, the T2DM subjects had a significantly higher insulin resistance index.

#### 1.2 Association of SNP+276 and T2DM

SNP+276 conformed to HWE in both the T2DM group and the NDM group (P = 0.72 and P = 0.34), respectively. The allele and genotype frequencies for SNP+276 are shown in Table S2. The allele frequencies for SNP+276 were 0.71 for G and 0.29 for T in the T2DM group. In the NDM group, the allele frequencies were 0.68 for G and 0.32 for T. The genotype frequencies for SNP+276 were 0.51 for GG, 0.40 for GT, and 0.09 for TT in the T2DM group. In the NDM group, the genotype frequencies were 0.45 for GG, 0.47 for GT, and 0.08 for TT. The genotype frequencies for SNP+276 showed no significant differences between the T2DM and NDM groups (χ^2^ = 1.59, P = 0.45). After adjusting age and gender, the genotype frequencies for SNP+276 also showed no significant differences between the T2DM and NDM groups (χ^2^ = 3.74, P = 0.15). In addition, the allele frequencies for SNP+276 showed no significant differences between the T2DM and NDM groups (χ^2^ = 0.65, P = 0.42, OR  = 0.87).

### 2. Meta-analysis result

#### 2.1 Flow of included studies

The initial search strategy to identify association studies for T2DM and SNPs in the adiponectin gene yielded a total of 262 potentially relevant references among all the databases, 146 of which were overlapping. After subsequent screening, 26 studies were identified for recruitment in the light of the inclusion criteria.

#### 2.2 Study characteristics

Table S3 and S4 show the 23 case-control studies for SNP+45, with a total of 4161 T2DM subjects and 3709 NDM subjects, and 11 case-control studies for SNP+276, with a total of 2533 T2DM subjects and 2212 NDM subjects, that were included in the analysis, respectively [16], [19], [20], [21], [22], [23], [24], [25], [26], [27], [28], [29], [30], [31], [32], [33], [34], [35], [36], [37], [38], [39], [40], [41], [42], [43]. All subjects were selected randomly without sex or age restriction. The diagnosis of T2DM followed the 1999 WHO Diabetes Criteria, except for one study that used the 1985 WHO criteria[23].

#### 2.3 Association of SNP+45 and SNP+276 with T2DM

**Figure 1:**
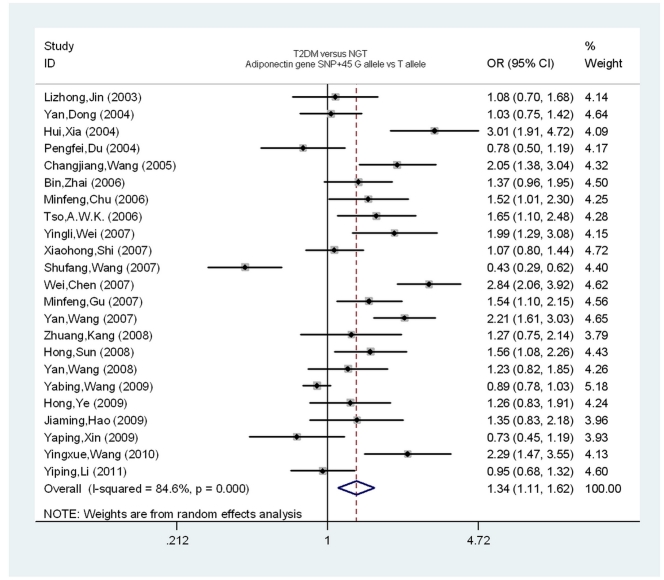
Individual and pooled ORs and 95% CI for SNP+45 (G vs.T) for all studies.

**Figure 2:**
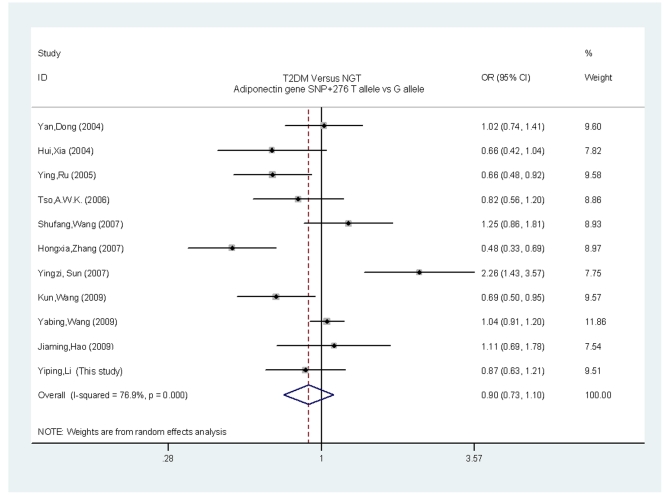
Individual and pooled ORs and 95% CI for SNP+276 (T vs.G) for all studies.

Fig. 1 and Fig. 2 show the ORs and 95% CI for the association of T2DM with SNP+45 and SNP+276, respectively, in individual studies. There was significant heterogeneity among the results of the individual studies, Pheterogeneity <0.01, I^2^ = 84.6% for SNP+45 and P_heterogeneity_ <0.01, I^2^ = 76.9% for SNP+276. Therefore, the random-effects model (I-V heterogeneity) was used to calculate the pooled ORs and 95% CI for both SNPs. Overall, there was a trend towards an increased risk of T2DM for the SNP+45G allele as compared with the SNP+45T allele (OR = 1.34, 95% CI = 1.11–1.62, P<0.01). However, there was no trend towards an increased risk of T2DM for the SNP+276T allele as compared with the SNP+276G allele (OR = 0.90, 95% CI = 0.73–1.10, P = 0.31).

#### 2.4 Publication bias

**Figure 3:**
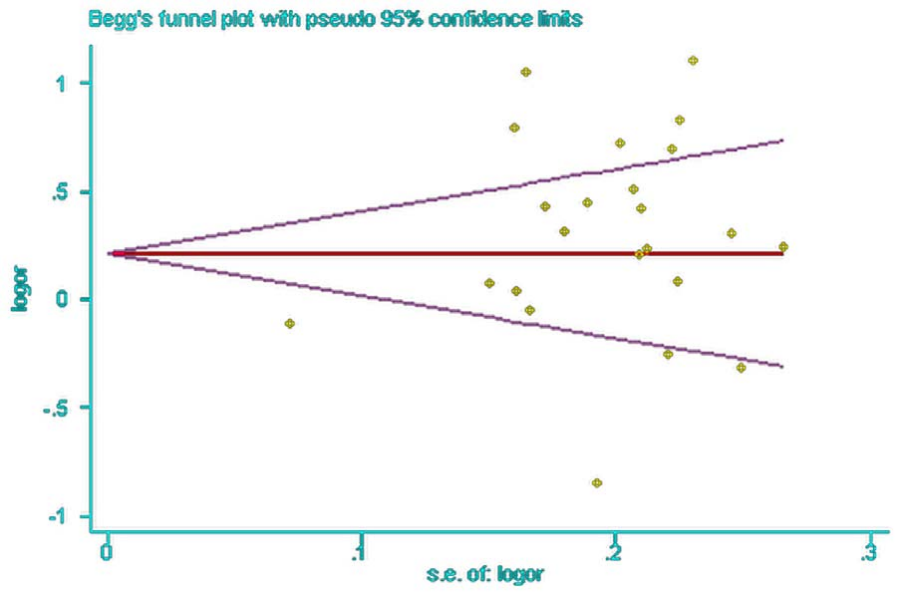
Begg’s funnel plot analysis for the comparison of the SNP+45 alleles. P value of Begg's test was 0.79 (continuity corrected).

**Figure 4:**
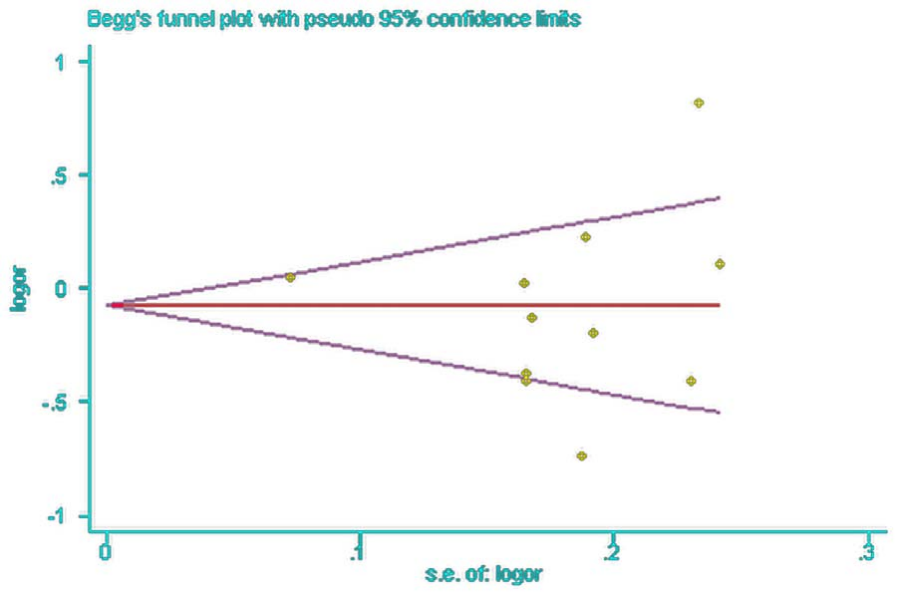
Begg's funnel plot analysis for the comparison of the SNP+276 alleles. P value of Begg's test was 0.76 (continuity corrected).

Begg's funnel plot and Egger's test were performed to determine whether the literature showed a publication bias. The Begg's funnel plots were symmetrical by visual inspection (Fig. 3 and Fig. 4), and neither the Begg's test nor the Egger's test suggested publication bias. The P-values for Egger's test were 0.07 (95% CI = −2.97 to 5.85) for SNP+45 and 0.60 (95% CI = −4.69 to 2.87) for SNP+276.

#### 2.5 Sensitivity analysis

A sensitivity analysis was performed by omitting one study at a time and calculating the pooled ORs for the remaining studies. This analysis showed that none of the individual studies influenced the pooled ORs, which ranged from 1.29 (95% CI = 1.08–1.55) to 1.41 (95% CI = 1.19–1.69) for SNP+45, and 0.85 (95% CI = 0.76–0.96) to 0.97 (95% CI = 0.88–1.06) for SNP+276. The sensitivity analysis indicated that the results of the meta-analysis were reliable and stable.

## Discussion

The adiponectin gene is located on human chromosome 3q27, where a region identified as a susceptibility locus for metabolic syndrome and T2DM has been reported, and is composed of three exons that span 17 kb[44], [45], [46], [47]. Two common SNPs in the adiponectin gene (SNP+45 and SNP+276) were determined to be associated with T2DM in the Japanese population by Hara et al[11]. They observed that Japanese subjects with the SNP+45GG genotype or SNP+276GG genotype had a significantly increased risk of T2DM. In the current study, the associations of the SNP+276 alleles and genotypes with T2DM were assessed separately, and no significant association of SNP+276 with T2DM was found. These results were similar to some previous studies of the Chinese Han population[19], [20], [23], [26], [37], but contradicted other studies that showed there was a significant association of SNP+276 with T2DM [32], [35]. The conflicting results indicated that a meta-analysis should be performed to determine whether these SNPs in the adiponectin gene were associated with T2DM in the Chinese Han population.

To the best of our knowledge, this is one of the largest systematic reviews of the literature that has used a meta-analysis to study the association between the SNP+45 and SNP+276 polymorphisms and T2DM in Chinese Han populations. The previous meta-analysis included eight papers for SNP+45 and four papers for SNP+276[48], whereas we combined our results with those of recently published studies, namely 23 papers for SNP+45 and 11 papers for SNP+276. As a result, we were able to conduct a comprehensive meta-analysis in which the number of studies was almost three times higher than in the previous analysis[48].

HWE is the principal law in population genetic studies. If a locus conforms with HWE, the samples are representative. In fifteen studies of SNP+45 and nine studies of SNP+276, the genotypes in both the T2DM and NDM groups conformed with HWE. Therefore, a further meta-analysis was performed in which only the studies that conformed with HWE were included. The further meta-analysis data showed that there was heterogeneity among the results of the individual studies, P_heterogeneity_ <0.01, I^2^ = 76.9% for SNP+45 and P_heterogeneity_ <0.01, I^2^ = 71.3% for SNP+276. The results from the application of the random-effect models revealed that subjects who carried the SNP+45G allele had a significantly increased risk of T2DM as compared with those who carried the SNP+45T allele (OR = 1.40; 95% CI, 1.16–1.68; P<0.01). However, there was no trend towards an increased risk of T2DM for the SNP+276T allele as compared with the SNP+276G allele (OR = 0.93; 95% CI, 0.76–1.14; P = 0.46). These results were similar to those of the initial meta-analysis in which all the studies were included.

Heterogeneity is a potential problem when interpreting the results of meta-analysis [49]. Our meta-analysis revealed significant between-study heterogeneity for SNP+45 (P_heterogeneity_ <0.01, I^2^ = 84.6%) and SNP+276 (SNP+276 P_heterogeneity_ <0.01, I^2^ = 76.9%). All of the subjects in the current meta-analysis study were Han Chinese but from different regions and the Han Chinese population is not a genetically homogenous group. Thus, this might explain the significant between-study heterogeneity. The differences in age and gender distributions among the included studies, and differences in sample content might also contribute to the heterogeneity.

In 2002, Hara *et al* first reported that the SNP45+GG genotype of the adiponectin gene was associated with T2DM in the Japanese population[11]. In 2005, Zacharova et al investigated the SNP+45 and SNP+276 polymorphisms of the adiponectin gene as predictors of the conversion from impaired glucose tolerance to T2DM in the STOP-NIDDM trial[50]. They observed that the SNP+45G allele was associated with a 1.8-fold increased risk of T2DM (95% CI, 1.12–3.00; P = 0.015) in the placebo group[50]. As a consequence, they concluded that the SNP+45G allele is a predictor of conversion to T2DM [50]. Many studies on the Chinese population also observed an association between the SNP+45G allele and T2DM [20], [21], [33], [38], [39]. These results agreed with our meta-analysis data (OR = 1.34; 95% CI, 1.11–1.62; P<0.01). Thus, we inferred that the SNP+45G allele might be a susceptibility allele for T2DM. However, the SNP+45 polymorphism, which is located within exon 2 of the adiponectin gene, does not alter the amino acid sequence of the adiponectin protein. Thus, rather than having a direct effect itself, SNP+45 might be in linkage disequilibrium with a polymorphism in one of the introns that destabilizes the pre-mRNA, results in reduced mRNA levels, and finally leads to pathophysiological effects[51]. Systematic screening of the intronic regions of the adiponectin gene to identify other SNPs should clarify why the SNP+45G allele is associated with T2DM in many studies and in our meta-analysis.

Hara et al. also reported that the SNP+276GG genotype of the adiponectin gene was associated with T2DM in the Japanese population[11]. However, we did not observe any association of SNP276 with T2DM in the current study. Many other studies also did not observe this association in the Chinese Han population[19], [20], [23], [26], [37]. In addition, the results of our meta-analysis showed no significant association between SNP+276 and T2DM (OR = 0.90; 95% CI, 0.73–1.10; P = 0.31). Possible explanations for the absence of an association with T2DM are: (a) the SNP+276 polymorphism in the adiponectin gene is not a candidate SNP for T2DM in the Chinese Han population; (b) the effect of SNP+276 might be masked by the summed effects of other unidentified genetic factors; (c) only 11 studies were included in our meta-analysis. Thus, a conclusive assessment of the possible association probably requires the inclusion of more studies and larger sample sizes. It was an interesting finding that the SNP+45 and SNP+276 are only 231 bp far away in adiponectin gene, however, only SNP+45 associated with T2DM in our meta-analysis. Wang et al. reported that SNP+45 just were partly in linkage disequilibrium with SNP+276 in Chinese Han[37]. Therefore, we assumed this could be possible one of causes of the divergence that only SNP+45 associated with T2DM in Han Chinese, but not SNP+276.

The results of our meta-analysis demonstrated that the SNP+45G allele might be a susceptibility allele for T2DM. However, we could not observe any association of SNP+276 with T2DM. As we know, T2DM is influenced by gene–environment interactions. In addition, many genetic factors, including the adiponectin gene, influence the occurrence of T2DM. Therefore, in order to understand the mechanisms underlying T2DM better, future research should be considered and carried out to explore the effects of gene–gene interactions, environmental factors, and individual genetic background.

## Materials and Methods

### 1. Association study between SNP+276 and T2DM

#### 1.1 Ethics statement

All participants gave written informed consent. The protocol was in accordance with the Helsinki Declaration, and was approved by the Institutional Review Boards of the Second People's Hospital of Yunnan Province.

#### 1.2 Subjects

The study included 202 patients (males = 121; females = 81) who were diagnosed with T2DM at the Second People's Hospital of Yunnan Province from December 2006 to September 2010. T2DM was confirmed using the criteria of the World Health Organization from 1999. Patients who were treated with insulin or an insulin secretagogue were excluded from the study. The nondiabetes group (NDM) included 143 subjects (males = 59; females = 84) who had no family history of diabetes mellitus and were recruited from an unselected population undergoing routine health checkups at the Second People's Hospital of Yunnan Province. Subjects with diabetes or impaired glucose tolerance were excluded from the NDM group on the basis of an oral glucose tolerance test. In addition, subjects with hypertension or coronary heart disease were also excluded from the study. All participants were ethnic Han by self report.

#### 1.3 Clinic measurements

Weight, height, waist circumference (WC), and hip circumference (HC) were measured by trained personnel in duplicate and the results were averaged. WC was measured midway between the lower rib margin and the iliac crest. HC was measured at the level of the greater trochanter. Body mass index (BMI) was calculated as weight (kg) divided by the square of height (m^2^). Waist to hip ratio (WHR) was calculated as WC divided by HC.

#### 1.4 Laboratory measurements

Venous blood samples were collected in the morning after the subjects had fasted for 12 hours. Fasting plasma glucose (FPG) was assayed by the glucose oxidase method using a HITACHI 7600-020 Automatic Analyzer. Total cholesterol (TC), high-density lipoprotein cholesterol (HDL-C), and triglycerides (TG) were determined by enzymatic methods with a HITACHI 7600-020 Automatic Analyzer. Low-density lipoprotein cholesterol (LDL-C) was calculated by using the Friedewald formula. Glycosylated hemoglobin (HbA1c) was determined by immunoturbidimetry with a HITACHI 7600-020 Automatic Analyzer. Fasting serum insulin was measured by radioimmunoassay with a Gamma counter. Insulin resistance was assessed by homeostatic model assessment (HOMA; insulin resistance index  =  [fasting glucose (mmol/L)×fasting insulin (µU/ml)]/22.5).

#### 1.5 Genotyping the SNP+276 polymorphisms in the adiponectin gene

Genomic DNA was extracted from peripheral lymphocytes by a standard hydroxybenzene–chloroform method. SNP+276 (rs1501299) was genotyped by the polymerase chain reaction-based restriction fragment-length polymorphism (PCR-RFLP) method. The PCR product was 745 bp and was digested with BmsI into fragments of 523 bp and 222 bp. The digested products were subjected to electrophoresis through a 2% agarose gel, stained with ethidium bromide, and visualized under ultraviolet light. Some of the PCR products were characterized by direct sequencing (3100 Genetic Analyzer; Applied Biosystems, Tokyo, Japan) using a BigDye Terminator v3.1 Cycle Sequencing Kit (Applied Biosystems, Foster City, CA, USA) after purification with Sephadex^TM^ G-50 (GE Healthcare, Piscataway, NJ, USA).

#### 1.6 Statistical analysis for the population and case-control study

The allele and genotype frequencies for SNP+276 were calculated by the direct-counting method. Hardy–Weinberg equilibrium (HWE) was tested in both the cases and the controls (http://ihg2.helmholtz-muenchen.de/cgi-bin/hw/hwa1.pl). A χ^2^ test was used to determine differences in allele and genotype frequencies between T2DM subjects and NDM subjects. The association of SNPs with T2DM was assessed by logistic regression after adjusting for age and gender. A P value of less than 0.05 was considered statistically significant.

### 2. Meta-analysis Study

#### 2.1 Search strategy

Our meta-analyses use the PRISMA statement as a guide. All of the studies included in the meta-analysis were studies of the association between the adiponectin SNPs and T2DM in Chinese populations. Sources included the MEDLINE, EMBASE, Chinese VIP, Chinese CNKI, and Chinese WanFang databases (up to February 2011). The search terms were as follows: ‘adiponectin’ OR ‘APM1’, ‘polymorphism’ AND ‘type 2 diabetes mellitus’, ‘China’ OR ‘Chinese’.

#### 2.2 Selection

To be included in the meta-analysis, studies had to meet all the following criteria: (a) an unrelated case-control design, (b) all subjects were Han Chinese, (c) available genotype and allele numbers or frequencies, (d) published in any language. PCR-single stranded conformational polymorphism (PCR-SSCP) or PCR-RFLP analysis and DNA sequencing were used to examine the mutations in the adiponectin gene. If there were multiple publications from the same group, only the most recent study was included in the analysis.

#### 2.3 Data extraction and study characteristics

The two authors who conducted the literature search (Yiping Li and Li Shi) also extracted the data from the studies independently. Any disagreement was adjudicated by consensus and by consulting two additional authors (Ying Zhang and Yufeng Yao). Author name, journal and year of publication, region of origin, diagnosis criteria for T2DM, size of case and control groups, and adiponectin SNP loci analyzed were collected. Sample sizes, genotype and allele numbers, and P values for HWE in the T2DM and NDM groups were summarized (Table S3 and Table S4).

#### 2.4 Statistical meta-analysis

Hardy–Weinberg equilibrium (HWE) was tested for all studies included (http://ihg2.helmholtz-muenchen.de/cgi-bin/hw/hwa1.pl). Then, we compared T2DM cases with controls by using the allele model (A/a) for each SNP. The odds ratios (ORs) and 95% confidence intervals (CI) were calculated to determine the differences in each SNP between the T2DM and NDM groups. The χ^2^-based Q statistic test was used to assess the between-study heterogeneity[52]. Heterogeneity was considered significant at a P value of <0.10. The inconsistency index I^2^ was also calculated to evaluate the amount of variation that was caused by heterogeneity rather than by chance: higher values of the index indicate the existence of heterogeneity[53]. Data were combined using both fixed-effects (Mantel–Haenszel) and random-effects (I-V heterogeneity) models. The fixed-effects model (if p>0.10) or the random-effects model (if p<0.10) was used to pool the results. From the individual ORs, a pooled OR was estimated, whose statistical significance was determined using the Z-test. Sensitivity analysis was performed to assess the stability of these results. Potential publication bias was estimated by Begg's funnel plot and Egger's linear regression test. P<0.05 was considered to represent a statistically significant publication bias[54], [55]. All P-values were two-sided. All meta-analysis was performed using Stata statistical software (STATA version SE-10.1; Stata Corporation, College Station, TX).

## Supporting Information

Table S1 Clinical characteristics of the subjects enrolled in the present study.
(DOCX)

Table S2 Comparison of genotypic and allelic distribution of SNP+45 and SNP+276 between type 2 diabetic and nondiabetic subjects.
(DOCX)

Table S3 Characteristcs of case-control studies included in a meta-analysis of the association between SNP+45 of adponectin gene and T2DM.
(DOCX)

Table S4 Characteristcs of case-control studies included in a meta-analysis of the association between SNP+276 of adponectin gene and T2DM.
(DOCX)
