# Supplementary material for: Association of Adiponectin SNP+45 and SNP+276 with Type 2 Diabetes in Han Chinese Populations: A Meta-Analysis of 26 Case-Control Studies
Source: PLoS One. 2011 May 11;6(5):e19686. doi: 10.1371/journal.pone.0019686 (PMC3092748; doi:10.1371/journal.pone.0019686)
Supplement: Table S1 — Clinical characteristics of the subjects enrolled in the present study. (DOCX) [file pone.0019686.s001.docx]

**Table S1 Clinical characteristics of the subjects enrolled in the present study (Data are mean±SD)**

|  | T2DM | NDM | P |
| --- | --- | --- | --- |
| N | 202 | 143 |  |
| Age(years) | 49.12±12.19 | 36.64±14.14 | <0.05 |
| Sex(male/female) | 121/81 | 59/84 | <0.05 |
| BMI(Kg/m^2^) | 24.52±3.26 | 21.45±3.42 | <0.05 |
| Waist Circumference(cm) | 88.33±9.66 | 76.84±11.17 | <0.05 |
| Hip circumference(cm) | 95.81±7.62 | 88.28±7.80 | <0.05 |
| WHR | 0.92±0.06 | 0.87±0.08 | <0.05 |
| TC(mmol/L) | 4.86±1.13 | 4.24±0.71 | <0.05 |
| HDL-C(mmol/L) | 1.17±0.29 | 1.33±0.30 | <0.05 |
| LDL-C(mmol/L) | 2.54±0.86 | 2.32±0.60 | <0.05 |
| FPG(mmol/L) | 7.91±2.23 | 4.31±0.68 | <0.05 |
| FIns(mU/L) | 12.62±8.17 | 13.25±8.46 | 0.49 |
| HbA1c(%) | 9.27±2.49 | 4.67±0.80 | <0.05 |
| HOMA | 4.53±3.60 | 2.52±1.63 | <0.05 |
