# Supplementary material for: Association of Adiponectin SNP+45 and SNP+276 with Type 2 Diabetes in Han Chinese Populations: A Meta-Analysis of 26 Case-Control Studies
Source: PLoS One. 2011 May 11;6(5):e19686. doi: 10.1371/journal.pone.0019686 (PMC3092748; doi:10.1371/journal.pone.0019686)
Supplement: Table S2 — Comparison of genotypic and allelic distribution of SNP+45 and SNP+276 between type 2 diabetic and nondiabetic subjects. (DOCX) [file pone.0019686.s002.docx]

**Table S2 Comparison of genotypic and allelic distribution of SNP+45 and SNP+276 between type 2 diabetic and nondiabetic subjects**

| SNP | Genotype[n(%)] | | | χ² | P | Alleles [n(%)] | | χ² | P | OR [95%CI] |
| --- | --- | --- | --- | --- | --- | --- | --- | --- | --- | --- |
| +45 ^a^ | TT | TG | GG |  |  | T | G |  |  |  |
| T2DM | 97(48.0) | 86(42.6) | 19(9.4) | 0.12 | 0.94 | 280(69.3) | 124(30.7) | 0.10 | 0.75 | 0.95 [0.68-1.32] |
| NDM | 67(46.9) | 61(42.7) | 15(10.5) |  |  | 195(68.2) | 91(31.8) |  |  |  |
| +276 | GG | GT | TT |  |  | G | T |  |  |  |
| T2DM | 103(51.0) | 81(40.1) | 18(8.9) | 1.59 | 0.45 | 287(71.0) | 117(29.0) | 0.65 | 0.42 | 0.87 [0.63-1.21] |
| NDM | 64(44.7) | 67(46.9) | 12(8.4) |  |  | 195(68.2) | 91(31.8) |  |  |  |

^a^ Allele and Genotype frequencies were cumulated from our previous studies (29)
