# Supplementary material for: Association of Adiponectin SNP+45 and SNP+276 with Type 2 Diabetes in Han Chinese Populations: A Meta-Analysis of 26 Case-Control Studies
Source: PLoS One. 2011 May 11;6(5):e19686. doi: 10.1371/journal.pone.0019686 (PMC3092748; doi:10.1371/journal.pone.0019686)
Supplement: Table S3 — Characteristcs of case-control studies included in a meta-analysis of the association between SNP+45 of adponectin gene and T2DM. (DOCX) [file pone.0019686.s003.docx]

**Table S3. Characteristics of case-control studies included in a meta-analysis of the association between SNP+45 of adiponectin gene and T2DM**

| Study ID | Author | year | region | Case | | | | | | control | | | | | |
| --- | --- | --- | --- | --- | --- | --- | --- | --- | --- | --- | --- | --- | --- | --- | --- |
|  |  |  |  | TT | TG | GG | T | G | HWE | TT | TG | GG | T | G | HWE |
| 001 | Lizhong,Jin | 2003 | Tianjin | 52 | 32 | 8 | 136 | 48 | 0.35 | 64 | 56 | 2 | 184 | 60 | 0.01 |
| 002 | Yan,Dong | 2004 | Shanghai | 104 | 71 | 20 | 279 | 111 | 0.14 | 98 | 74 | 15 | 270 | 104 | 0.84 |
| 003 | Hui,Xia | 2004 | jiangsu | 8 | 46 | 24 | 62 | 94 | 0.04 | 39 | 35 | 11 | 113 | 57 | 0.48 |
| 004 | Pengfei,Du | 2004 | Shanghai | 74 | 27 | 12 | 175 | 51 | <0.01 | 55 | 47 | 6 | 157 | 59 | 0.32 |
| 005 | Changjiang,Wang | 2005 | Anhui | 56 | 68 | 19 | 180 | 106 | 0.82 | 67 | 40 | 5 | 174 | 50 | 0.75 |
| 006 | Bin,Zhai | 2006 | Beijing | 103 | 69 | 23 | 275 | 115 | 0.04 | 78 | 57 | 4 | 213 | 65 | 0.07 |
| 007 | Minfeng,Chu | 2006 | Beijing | 73 | 53 | 9 | 199 | 71 | 0.88 | 86 | 37 | 6 | 209 | 49 | 0.44 |
| 008 | Tso,A.W.K. | 2006 | South China | 69 | 79 | 10 | 217 | 99 | 0.04 | 67 | 29 | 8 | 163 | 45 | 0.07 |
| 009 | Yingli,Wei | 2007 | Ninxia | 39 | 48 | 13 | 126 | 74 | 0.77 | 58 | 40 | 3 | 156 | 46 | 0.21 |
| 010 | Xiaohong,Shi | 2007 | Beijing and Heilongjiang | 89 | 78 | 12 | 256 | 102 | 0.35 | 148 | 112 | 20 | 408 | 152 | 0.85 |
| 011 | Shufang,Wang | 2007 | Shangdong | 90 | 36 | 12 | 216 | 60 | 0.01 | 48 | 64 | 20 | 160 | 104 | 0.86 |
| 012 | Wei,Chen | 2007 | Henan | 20 | 94 | 54 | 134 | 202 | 0.03 | 68 | 60 | 22 | 196 | 104 | 0.15 |
| 013 | Minfeng,Gu | 2007 | jiangsu | 67 | 94 | 18 | 228 | 130 | 0.07 | 74 | 62 | 8 | 210 | 78 | 0.28 |
| 014 | Yan,Wang | 2007 | Guangdong | 80 | 92 | 28 | 252 | 148 | 0.85 | 122 | 72 | 6 | 316 | 84 | 0.23 |
| 015 | Zhuang,Kang | 2008 | Yunnan | 46 | 31 | 9 | 123 | 49 | 0.29 | 35 | 29 | 1 | 99 | 31 | 0.07 |
| 016 | Hong,Sun | 2008 | Hunan | 126 | 115 | 14 | 367 | 143 | 0.06 | 76 | 40 | 4 | 192 | 48 | 0.65 |
| 017 | Yan,Wang | 2008 | Hubei | 116 | 96 | 16 | 328 | 128 | 0.52 | 45 | 36 | 2 | 126 | 40 | 0.09 |
| 018 | Yabing,Wang | 2009 | Shanghai | 480 | 362 | 74 | 1322 | 510 | 0.62 | 483 | 389 | 98 | 1355 | 585 | 0.14 |
| 019 | Hong,Ye | 2009 | Fujian | 68 | 52 | 11 | 188 | 74 | 0.81 | 59 | 42 | 4 | 160 | 50 | 0.29 |
| 020 | Jiaming,Hao | 2009 | Hainan | 44 | 44 | 18 | 132 | 80 | 0.23 | 28 | 24 | 6 | 80 | 36 | 0.80 |
| 021 | Yaping,Xin | 2009 | Henan | 14 | 27 | 23 | 55 | 73 | 0.27 | 15 | 21 | 36 | 51 | 93 | <0.01 |
| 022 | Yingxue,Wang | 2010 | Shangdong | 38 | 47 | 15 | 123 | 77 | 0.94 | 60 | 37 | 3 | 157 | 43 | 0.34 |
| 023 | Yiping,Li | 2011 | Yunnan | 97 | 86 | 19 | 280 | 124 | 0.99 | 67 | 61 | 15 | 195 | 91 | 0.84 |

HWE, Hardy–Weinberg equilibrium.*P < 0.05 indicated inconsistence with Hardy–Weinberg equilibrium.
