# Supplementary material for: Association of Adiponectin SNP+45 and SNP+276 with Type 2 Diabetes in Han Chinese Populations: A Meta-Analysis of 26 Case-Control Studies
Source: PLoS One. 2011 May 11;6(5):e19686. doi: 10.1371/journal.pone.0019686 (PMC3092748; doi:10.1371/journal.pone.0019686)
Supplement: Table S4 — Characteristcs of case-control studies included in a meta-analysis of the association between SNP+276 of adponectin gene and T2DM. (DOCX) [file pone.0019686.s004.docx]

**Table S4. Characteristics of case-control studies included in a meta-analysis of the association between SNP+276 of adiponectin gene and T2DM**

| NO. | Author | year | region | Case | | | | | | Control | | | | | |
| --- | --- | --- | --- | --- | --- | --- | --- | --- | --- | --- | --- | --- | --- | --- | --- |
|  |  |  |  | GG | TG | TT | G | T | HWE | GG | TG | TT | G | T | HWE |
| 001 | Yan,Dong | 2004 | Shanghai | 104 | 71 | 16 | 279 | 103 | 0.44 | 100 | 73 | 13 | 273 | 99 | 0.95 |
| 002 | Hui,Xia | 2004 | jiangsu | 32 | 41 | 5 | 105 | 51 | 0.09 | 26 | 46 | 13 | 98 | 72 | 0.32 |
| 003 | Ying,Ru | 2005 | Anhui | 164 | 101 | 11 | 429 | 123 | 0.37 | 69 | 59 | 13 | 197 | 85 | 0.94 |
| 004 | Tso,A.W.K. | 2006 | Honggang | 82 | 59 | 17 | 223 | 93 | 0.21 | 49 | 40 | 15 | 138 | 70 | 0.16 |
| 005 | Shufang,Wang | 2007 | Shangdong | 56 | 76 | 6 | 188 | 88 | <0.01 | 68 | 56 | 8 | 192 | 72 | 0.43 |
| 006 | Hongxia,Zhang | 2007 | Shangdong | 44 | 93 | 61 | 181 | 215 | 0.45 | 18 | 20 | 60 | 56 | 140 | <0.01 |
| 007 | Yingzi, Sun | 2007 | Shanxi | 35 | 31 | 10 | 101 | 51 | 0.46 | 90 | 39 | 5 | 219 | 49 | 0.76 |
| 008 | Kun,Wang | 2009 | Tianjin | 110 | 75 | 11 | 295 | 97 | 0.70 | 76 | 71 | 18 | 223 | 107 | 0.82 |
| 009 | Yabing,Wang | 2009 | Shanghai | 451 | 397 | 66 | 1299 | 529 | 0.09 | 496 | 398 | 72 | 1390 | 542 | 0.52 |
| 010 | Jiaming,Hao | 2009 | Hainan | 46 | 42 | 18 | 134 | 78 | 0.13 | 26 | 24 | 8 | 76 | 40 | 0.52 |
| 011 | Yiping,Li | 2011 | Yunnan | 103 | 81 | 18 | 287 | 117 | 0.72 | 64 | 67 | 12 | 195 | 91 | 0.34 |

HWE, Hardy–Weinberg equilibrium.*P < 0.05 indicated inconsistence with Hardy–Weinberg equilibrium.
